# Microstructural Evolution, Hardness and Wear Resistance of WC-Co-Ni Composite Coatings Fabricated by Laser Cladding

**DOI:** 10.3390/ma17092116

**Published:** 2024-04-30

**Authors:** Gibeom Kim, Yong-Chan Kim, Jae-Eock Cho, Chang-Hee Yim, Deok-Su Yun, Tae-Gyu Lee, Nam-Kyu Park, Rae-Hyung Chung, Dae-Geun Hong

**Affiliations:** 1 Graduate Institute of Ferrous & Eco Materials Technology, Pohang University of Science & Technology (POSTECH), 77 Cheongam-Ro, Nam-Gu, Pohang-si 37673, Gyeongsangbuk-do, Republic of Korea; ggbeom@postech.ac.kr (G.K.); dady1@postech.ac.kr (J.-E.C.); chyim@postech.ac.kr (C.-H.Y.); 2 Research Institute of Industrial Science and Technology (RIST), 67 Cheongam-Ro, Nam-Gu, Pohang-si 37673, Gyeongsangbuk-do, Republic of Korea; yongchankim@rist.re.kr (Y.-C.K.); jungrh7@hanmail.net (R.-H.C.); 3 SungWook Co., Ltd., 74-43, Chaesin1gongdan-gil, Yeongcheon-si 38899, Gyeongsangbuk-do, Republic of Korea; dukho861012@daum.net (D.-S.Y.); taeng2a@naver.com (T.-G.L.); kyyu1124@gmail.com (N.-K.P.)

**Keywords:** continuous caster rolls, laser cladding, WC-Co alloy powder, hardness, wear resistance, laser powder, cladding speed, powder feeding rate

## Abstract

This study investigated how process parameters of laser cladding affect the microstructure and mechanical properties of WC-12Co composite coating for use as a protective layer of continuous caster rolls. WC-Co powders, WC-Ni powders, and Ni-Cr alloy powder with various wear resistance characteristics were evaluated in order to determine their applicability for use as cladding materials for continuous caster roll coating. The cladding process was conducted with various parameters, including laser powers, cladding speeds, and powder feeding rates, then the phases, microstructure, and micro-hardness of the cladding layer were analyzed in each specimen. Results indicate that, to increase the hardness of the cladding layer in WC-Co composite coating, the dilution of the cladding layer by dissolution of Fe from the substrate should be minimized, and the formation of the Fe-Co alloy phase should be prevented. The mechanical properties and wear resistance of each powder with the same process parameters were compared and analyzed. The microstructure and mechanical properties of the laser cladding layer depend not only on the process parameters, but also on the powder characteristics, such as WC particle size and the type of binder material. Additionally, depending on the degree of thermal decomposition of WC particles and evolution of W distribution within the cladding layer, the hardness of each powder can differ significantly, and the wear mechanism can change.

## 1. Introduction

During the continuous casting of steel, foot rolls and lateral rolls are employed in the zero-segment zone of the slab caster to guide the partially solidified slab. The surface coating quality of these rolls greatly influences both their service life and the quality of the produced slabs [1]. These rolls are exposed to high stress and high temperatures between 1000 °C and 1200 °C, and to continuous thermal cycling by water coolant sprayed from external devices [2,3]. These conditions can cause oxidation, surface degradation and thermal cracking in the cast roll and thereby reduce its service life [4,5]. To avoid these problems, the rolls are coated with materials with which to increase their surface hardness, wear resistance, and corrosion resistance [6,7].

The conventional roll coating for continuous caster rolls consists mainly of arc welding and thermal spray. However, arc welding is not suitable when metal abrasion is severe and it has the disadvantage of reduced mechanical strength and corrosion resistance at high temperatures [5,8]. In contrast, thermal spray coating can degrade mechanical properties at the interface of the coating layer due to the characteristics of mechanical bonding [9]. Alternative methods are being sought.

Laser cladding is a roll-coating method that can be an effective technique to solve these corrosion and wear problems, and to increase the bonding strength between the coating layer with base material. A powder is sprayed on the surface of a substrate, and the laser is simultaneously irradiated to melt the powder to form a coating layer [1,10,11]. Laser cladding achieves good metallurgical bonding, lower dilution of the cladding layer, and lower distortion than conventional methods, such as arc welding and thermal spraying [12,13], but involves rapid temperature changes, which affect the mechanical properties and dimensional stability of the cladding layer. These rapid temperature changes can finally cause excessive heat-affected zones, cracks, and pore defects [14,15,16].

Steels are increasingly used in severe environments, such as those of extremely high or low temperature and high humidity, and this change has increased demands for quenching-and-partitioning steel, advanced multi-phase steel, and advanced high-strength steel [17]. The production of these steels by continuous casting increases the stresses on the caster rolls, and the corrosive environment occurs around them due to the chemical reaction between moisture and the mold flux supplied to the continuous caster. These problems can be addressed by using laser cladding process and materials that have appropriate mechanical properties, wear behavior and corrosion resistance. Laser cladding processes employing hard materials such as tungsten carbide (WC) and powders containing binder metals like Co and Ni have been reported to achieve excellent wear resistance and corrosion resistance. The addition of WC particles causes notable changes in microstructure and increases the microhardness, tensile strength and elongation of Ni-Cu and WC-12Co/Ni-Cu alloys [18,19,20]. WC-12Co-based powders have been utilized to improve these properties in Ni-Cu alloys and Inconel 718 alloys. WC and Co-Cu compounds are beneficial to wear resistance. However, the wear resistances of various cladding materials have not been compared. Process parameters also affect the properties of the laser cladding coating layers [21,22,23]. Appropriate adjustments of scanning speed *S* and laser power P can provide a uniform and defect-free microstructure in the cemented carbides. However, few studies have considered how the process parameters of laser cladding affect microstructural evolution and the mechanical properties of the cladding layer for application to continuous caster roll-coating technology.

Studies of laser cladding for continuous casting roll coating application have included use of powders that consist mainly of Fe or Ni [2,24], the addition of oxide or flux into cladding materials [14,25], and the optimization of the process parameters of laser cladding [1]. In this study, we performed a fundamental study of the microstructure, mechanical properties and wear resistance of coatings composed of WC-Co and WC-Ni composites according to the process parameters of laser cladding, and powder types. The WC-Co and WC-Ni composite coating powders have been widely applied for mold cavities, automotive brake discs, drilling tools, cutting cutlery, and wear-resistant parts due to their high hardness, wear resistance, oxidation resistance, and corrosion resistance [26,27,28]. Thus, this study tested the use of WC-Co powder for application in the laser cladding process in order to optimize roll coating for continuous casting, and investigated the effects of laser-cladding process parameters on microstructure and mechanical properties of the cladding layer. For comparison, WC-Ni powder, and Ni alloy powder were also tested as cladding materials, and specimens were laser-cladded under specific process parameters. Subsequently, their micro-hardness and wear resistance were evaluated.

## 2. Experimental

### 2.1. Materials

S45C steel was used as the coating of the continuous caster roll. This steel has similar mechanical properties to those of SCM 440, which is one of the Cr-Mo alloy steels that is generally used as the base material of continuous caster rolls [2,5]. We chose S45C because the aim of the study is to evaluate changes in microstructure and mechanical properties according to laser cladding process parameters and powder types for roll coating applications. Its chemical composition (wt%) is 0.42~0.48 C, 0.15~0.35 Si, 0.6~0.9 Mn, *p* < 0.03, *S* < 0.035. Four cladding powders were used: two types of WC-Co, one type of WC-Ni, and one type of Ni-based alloy powder (Table 1).

Powder A (Co-added) and powder D (Ni-based) are alloy powders that have been commercialized for laser cladding process. It should be noted that commercial powders such as powder A and powder D are utilized for specific purposes and have different particle sizes. Powder B (Co-added) and powder C (Ni-added) have been developed as potential replacements for powder A and powder D, respectively, ensuring that the particle sizes of powders A and B, as well as powders C and D, are comparable. It should also be emphasized that powder C is a blend of similarly sized nickel alloy powder and WC particles.

Coating powders that include WC were classified into Co-added and Ni-added types according to the type of binder metal [9]. Due to the characteristics of rolls for which wear resistance and impact resistance are important, WC-Co-based coating powders have been widely applied, and laser cladding layers produced using WC-12Co-based powders show high impact resistance and dense coating.

WC-12Co coating powder is widely used as a material with which to coat cast rolls in casting, and to coat pinch rolls in hot rolling [29,30]. The effect of Co content is a trade-off, one in which decreasing Co content increases the wear resistance, but decreases the impact resistance; therefore, Co content should be optimized according to the required coating performance. Powders that include Ni (C and D) have lower mechanical properties than powders that include Co, but have better resistance to corrosion and oxidation. Therefore, Ni-based metal powder was used as a substitute for Co-based powder in a corrosion environment in which Co metal is difficult to use.

**Figure 1:**
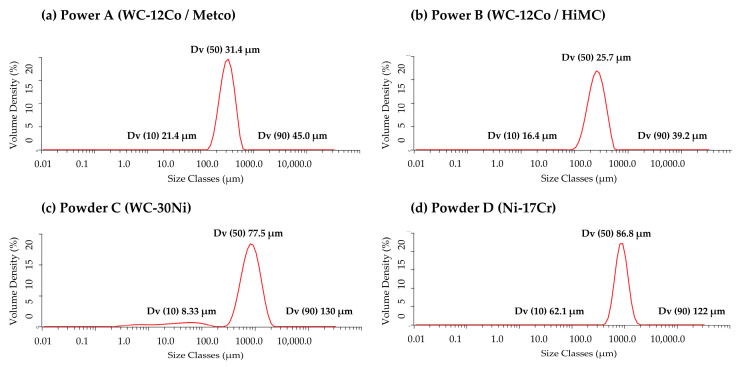
Particle size distribution of cladding powders.

Particle size distributions differed among the cladding powders (Figure 1). All of these powders exhibit a Gaussian distribution, and the median particle size (Dv50) were relatively fine in in powders A (31.4 um) and B (25.7 um), and coarse in powders C (77.5 um) and D (86.8 um). Particle size for powder has a significant effect on melting and solidification, and is therefore an important factor in the laser cladding process. The impacts of the particle sizes for the four powders on the microstructure and wear resistance will be discussed in Section 3.5.

**Figure 2:**
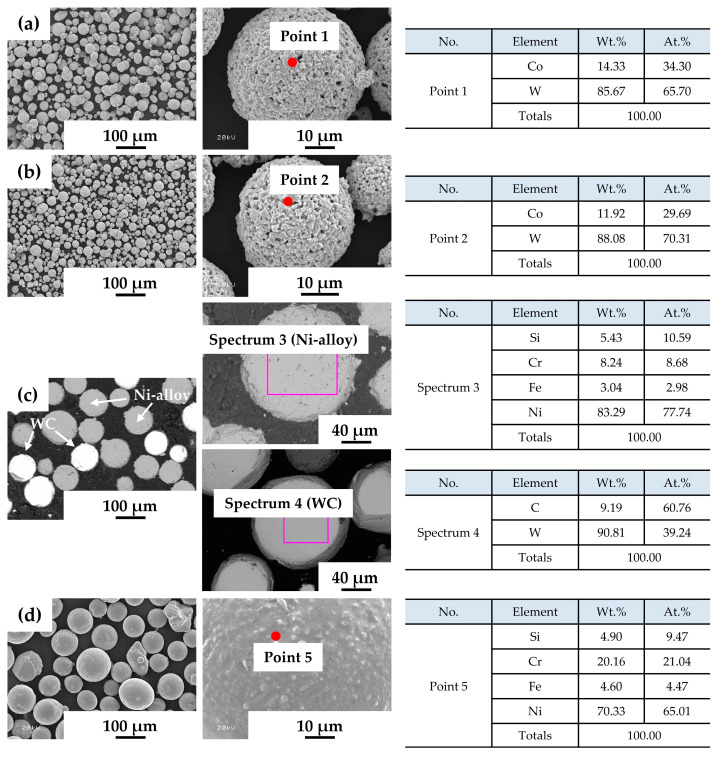
Morphology and chemical composition of cladding powders: (**a**) powder A (WC-12Co, Metco), (**b**) powder B (WC-12Co, HiMC); (**c**) powder C (WC-30Ni), (**d**) powder D (Ni-Cr alloy).

The characteristics of the particles and grains differed among the powders (Figure 2). Grains of powder A were polygonal, with a porous structure in which W and Co elements were mixed. Grains of powder B had slightly higher W contents and were smaller than Powder A. The particles of the powders are divided into Ni-alloy and WC particles; the Ni-alloy contains about 83 wt% Ni, while the WC particles have a dense structure and contain only W and C elements. The grains of powder D were densely spherical, with almost no pores and with generally uniform alloy compositions and Cr-rich phase distributed throughout an Ni-rich matrix.

### 2.2. Laser Cladding Process

The laser cladding experiment was performed using a 10 kW fiber-coupled diode laser (LDF 10000-60, Laserline, Mülheim-Kärlich, Germany) that has a tunable wavelength from 980 to 1080 nm and a laser spot size of 5.5 mm. The S45C steel substrate was 100 mm in width and height, and 20 mm in thickness. A powder-supply system equipped with coaxial and cooling systems was performed by using a coaxial nozzle attached to a robot arm (IRB 6700-6DOF, ABB, Zurich, Switzerland) as a powder disk supply principle (GTV, Luckenbach, Germany). Ar gas (>99.99%) was used at flow rates of 20 g/min as the shielding gas, and of 5 g/min as the carrier gas. After the laser cladding process, the specimen was allowed to cool passively to air temperature.

To analyze how process parameters affected the properties of the cladding layer, the experiments were conducted by using powder A with various laser power *P*, cladding speed *S*, powder feeding rate *R* at the same offset (Table 2, Specimen 1–10). Subsequently, the geometry of the cladding layer, the microstructure, and the micro-hardness were analyzed.

The mechanical properties and wear resistance of the laser-cladded specimens with the same process parameter that had been fabricated using WC-Co powders, WC-Ni powders, and Ni-Cr alloy powders were compared (Table 3, Specimen nos. 11–14). Each powder had different physical properties, and the coating characteristics varied with process parameters. However, in the context of this section focusing on the influence of powder type, the mechanical properties and wear characteristics, which were observed using different powders rather than laser cladding process parameters, were evaluated under the same process parameters.

### 2.3. Microstructure Analysis and Property Test

**Figure 3:**
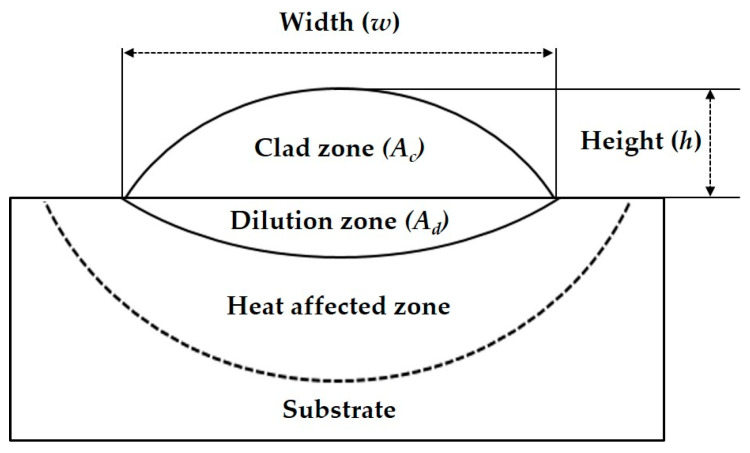
A cross section schematic of single cladding track.

After the laser cladding process, each specimen was cut in the cross-section. Then, the exposed surface was polished and observed using an optical microscope (OM, DSX-HRSU, Olympus, Tokyo, Japan) and a scanning electron microscopy–energy dispersive spectroscopy (SEM-EDS, JSM-7100F, JEOL, Tokyo, Japan) for analysis of morphology and phase. Each cladding track could be divided into several zones (Figure 3). To analyze the geometrical characteristics of the cladding layer, its height (*h*), width (*w*), and clad area (A_c_) above the substrate surface, and dilution area (A_d_) below the substrate surface were measured in OM images.

The *w*/*h* aspect ratio and dilution rate (*D*) were calculated using these geometrical dimensions. *D* is the mixing ratio between the cladding materials and substrate and is calculated from Equation (1) [31,32].

(1) $$D=\frac{A_{d}}{A_{c}{+A}_{d}}$$

Phases of the laser cladding specimen were identified using X-ray diffraction (XRD, D8 Advance-Davinci, Bruker, Billerica, MA, USA) measurement with Cu Kα radiation at 40 mA and 40 kV. XRD patterns were obtained at a scan speed of 0.3 s/step and a step size of 0.02° in the range between 15° and 105°.

The microhardness of the specimen was measured at intervals of about 200 to 500 μm across the interface between the cladding layer and the substrate, avoiding regions that included only WC particles. A Vickers hardness tester (HM-220, Miyutoyo, Kawasaki, Japan) was used under conditions of HV0.3, loading time of 10 s, and holding time of 10 s.

For comparison, Specimens 11–14 were fabricated using specific powders under the same process parameters, then subjected to a wear resistance test that was conducted according to ASTM G99-17 [33], using a ball-on-disk type tester that rubs a spherical specimen of a reference material against a disk-shaped specimen of the test material. The reference material was a 12.7 mm SiC, and the test specimen was a 31.8 mm diameter disc. The test parameters were 30 N normal load, 1000 rpm rotation speed, 3600 s duration, and room temperature.

## 3. Results and Discussion

### 3.1. Effect of Processing Parameters on the Cladding Layer

**Figure 4:**
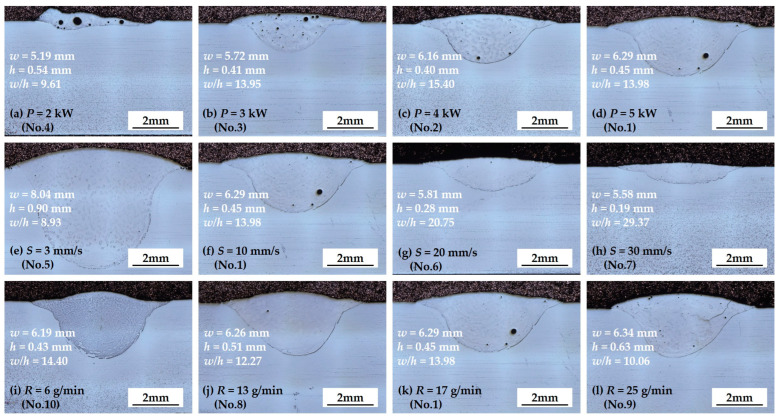
OM images of cross section of laser cladding specimen for various laser powers (*P*), cladding speeds (*S*), and powder feeding rates (*R*) in the specimen numbers (no.) defined in Table 2.

**Figure 5:**
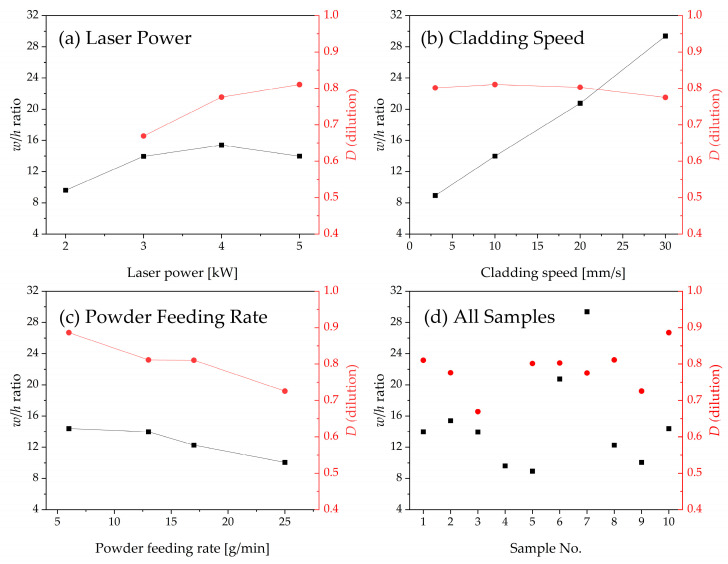
Geometry of cladding layer with various process parameters for powder A: (**a**) laser power (*P*); (**b**) cladding speed (*S*); (**c**) powder feeding rate (*R*); (**d**) all specimens.

The cross-section images of the specimens measured by OM were merged to demonstrate the change of the cladding layer according to changes in process parameters (Figure 4). The dimensions of the specimens were used to compare the *w*/*h* ratio and *D* of the cladding layer (Figure 5).

As laser power *P* increased, the *w* of the cladding layer increased because the energy input from the irradiated laser applied to the cladding powder and substrate increased. At *P* = 3 kW (specimen 3), the cladding layer had *w* = 5.72 mm and *h* = 0.41 mm. At *P* = 4 kW (specimen 2), the cladding had *w* = 6.16 mm and *h* = 0.40 mm. In contrast, at *P* = 2 kW, the shape of the cladding layer became non-uniform (Figure 4a) because the irradiated energy was not sufficient to totally melt the cladding powder. Therefore, *D* was not calculated for the specimen (No. 4) that had been treated with *P* = 2 kW.

As *P* increased, *w*/*h* ratio exhibited a slight increase until *P* reached 4 kW. Beyond this point, *w*/*h* ratio tended to decrease as *P* exceeded 4 kW. Concurrently, *D* continued to increase throughout the range (Figure 5a). The increase of *P* could provide sufficient energy with which to melt the cladding powders and increase the interaction time between cladding materials and substrate. An increase in *P* caused a decrease in the number of pores that formed during the solidification of cladding layer [34,35].

As cladding speed *S* increased, both *w* and *h* tended to decrease (Figure 5b). *S* = 3 mm/s had *w* = 8.04 mm and *h* = 0.9 mm (specimen 5), while *S* = 30 mm/s had *w* = 5.58 mm and *h* = 0.19 mm (specimen 7). Variation in *S* also affected the geometry of the cladding layer. At constant *P*, as *S* increased, the laser energy irradiated to a specific spot per unit time to the cladding powder and substrate decreased. Therefore, *w*/*h* increased and *D* decreased slightly. Specimen 7, with the fastest *S* = 30 mm/s had *w*/*h* = 29.37 and *D* = 0.78.

Powder feeding rate *R* also affected the geometry of the cladding layer (Figure 5c). As *R* increased, *h* increased slightly, and *w* did not change significantly. The smallest *R* = 6 g/min had *w* = 6.19 mm and *h* = 0.43 mm, and the highest *R* = 25 g/min had *w* = 6.34 mm and *h* = 0.63 mm. As *R* increased, the *w*/*h* ratio and *D* decreased significantly (Figure 5c). These results occur because the laser energy applied to the substrate and powder decreased as *R* increased. When *P* and *S* are constant, an increase in the amount of powder injected into the cladding layer decreased the laser energy absorbed, consequently reducing the interaction time between irradiated laser and substrate [36]. Specimen 3, which had low *P* and relatively high *R* showed the lowest *D*, and specimen 10, which had the smallest *R* had the largest *D* (Table 2 and Figure 5d).

In contrast, when multi-track laser cladding is performed, the *w*/*h* aspect ratio is a critical factor that causes the inter-run pores between the overlapped clad [37]; this ratio is affected by process parameters such as *S* and *R*. In particular, the pores frequently form in the area in which adjacent tracks overlap, and these pores appeared when the single track has *w*/*h* < 5 [38]. All specimens in this study satisfied *w*/*h* > 5. Additionally, excessive dilution and compositional non-homogeneities could lead to defects in laser cladding [39].

### 3.2. Microstructure Characteristics

SEM images were obtained in order to compare the microstructure of the cladding layer for powder A according to changes in processing parameters. To investigate the microstructural differences across different areas of laser cladding layer, SEM images of both the top and bottom regions were analyzed. The laser cladding process induces non-equilibrium solidification caused by a fast cooling rate of about 10^6^ K/s [40], and the action of the high-energy laser beam, meaning that the microstructure varies spatially for each specimen.

**Figure 6:**
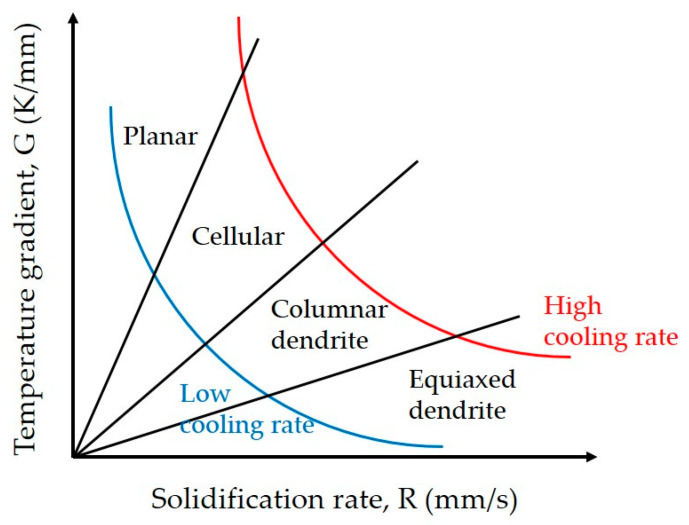
Effects of solidification rate and temperature gradient on the solidified microstructure.

The microstructure formed during the laser cladding process depends on the temperature gradient (G) and the solidification rate (R) (Figure 6) [41]. As the G/R ratio increases, the morphology of the microstructure is transformed from equiaxed dendrites into columnar dendrites, cellular crystals, and planar crystals. The cooling rate, represented by G × R, influences the grain size, with a higher cooling rate leading to finer grains.

**Figure 7:**
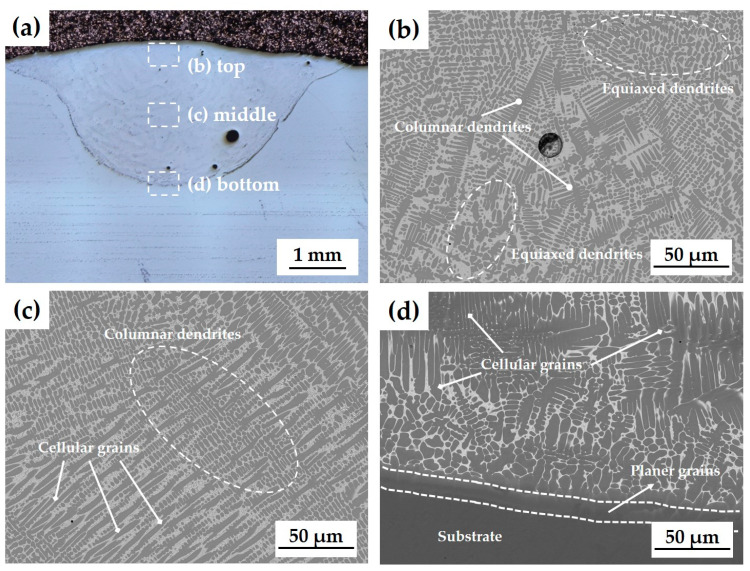
(**a**) Cross-section image of laser cladding layer for specimen 1 and microstructure of each region: (**b**) top; (**c**) middle; (**d**) bottom.

**Figure 8:**
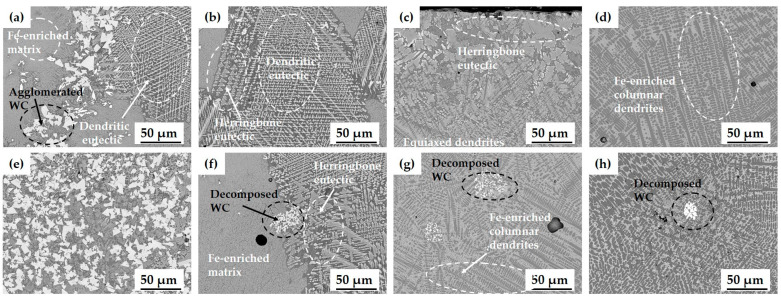
Microstructure of cladding layer with various laser powers (*P*) for powder A: (**a**) 2 kW (top); (**b**) 3 kW (top); (**c**) 4 kW (top); (**d**) 5 kW (top); (**e**) 2 kW (bottom); (**f**) 3 kW (bottom); (**g**) 4 kW (bottom); (**h**) 5 kW (bottom).

**Figure 9:**
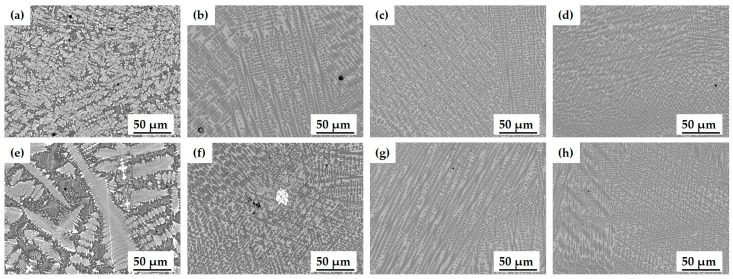
Microstructure of cladding layer with various cladding speeds (*S*) for powder A: (**a**) 3 mm/s (top); (**b**) 10 mm/s (top); (**c**) 20 mm/s (top); (**d**) 30 mm/s (top); (**e**) 3 mm/s (bottom); (**f**) 10 mm/s (bottom); (**g**) 20 mm/s (bottom); (**h**) 30 mm/s (bottom).

**Figure 10:**
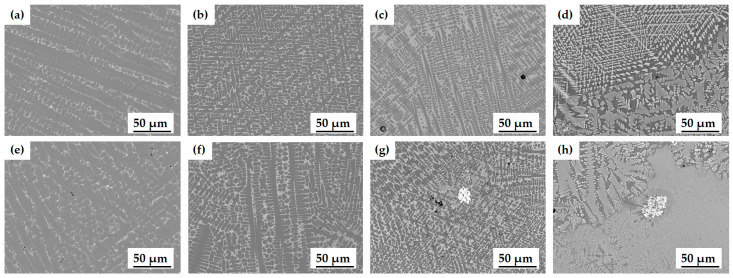
Microstructure of cladding layer with various powder feeding rates (*R*) for powder A: (**a**) 6 g/min (top); (**b**) 13 g/min (top); (**c**) 17 g/min (top); (**d**) 25 g/min (top); (**e**) 6 g/min (bottom); (**f**) 13 g/min (bottom); (**g**) 17 g/min (bottom); (**h**) 25 g/min (bottom).

During the solidification process, the thermal distribution in the cladding layer decreases from the bottom to the top surface in terms of the G/R ratio, whereas the cooling rate (G × R) increases [42]. Consequently, the G value at the top surface of the cladding layer is lower, and the *R* value is higher, resulting in a relatively small G/R ratio. This change predominantly facilitates the formation of columnar and equiaxed dendrites (Figure 7b). In contrast, towards the substrate interface at the bottom of the cladding layer, G is higher, and *R* is lower, leading to a larger G/R ratio and a predominance of planar and cellular grains (Figure 7d). The distinctive differences in the microstructure of the cladding layer between the top and bottom regions are observed throughout Figure 8, Figure 9 and Figure 10. The microstructure in the top region of the cladding layer develops columnar dendrites and exhibits relatively finer grains.

WC particles with diameter ≤ 10 μm in the cladding powder were agglomerated or thermally decomposed due to the action of laser energy and high temperature in the molten pool (Figure 8a,e) [43]. At *P* = 2 kW, images show WC particles that were not fully integrated and decomposed, Fe-enriched matrix phase, dendritic eutectic structure, and herringbone eutectic structure. As *P* was increased, the number of WC particles decreased. At *P* = 4 kW, Fe-enriched matrix phase grew, and Fe-enriched columnar dendrites formed (Figure 8c,g). Herringbone eutectic structure still occurred in the upper part of specimen. At *P* = 5 kW, decomposed WC particles and Fe-enriched matrix phases formed, and Fe-enriched columnar dendrites became the main microstructure of the entire area; the herringbone eutectic structure was not observed (Figure 8d,h). The microstructure of each specimen was affected by the process parameters because the laser cladding process entails numerous coupled factors, including process parameters, substrate and cladding materials.

*S* affected the microstructure of the cladding layer (Figure 9). At excessively high *S*, the cladding powder did not melt sufficiently; and at excessively low *S*, the cladding layer could overheat. At *S* = 3 mm/s, a herringbone eutectic structure grew up over the entire area, and the Fe-enriched matrix phase appeared around this structure. As *S* increased, the herringbone eutectic structure disappeared. The Fe-enriched columnar dendrite structure increases, and the size of the columnar dendrite became increasingly fine due to the relatively fast cooling rate. These results are similar to those of other studies [44,45], and the increase in *S* causes a decrease in the time available for the laser to react with the cladding material, so heat input decreases. Consequently, the change in temperature gradient and increased cooling rate affect the microstructure and phase composition of the cladding layer.

In contrast, as *R* increased, the number of Fe-enriched columnar dendrites decreased (Figure 10), while dendritic eutectic structure and herringbone eutectic structure increased. At *R* = 25 g/min, decomposed WC particles were frequently observed because the powder included a relatively large number of WC particles. Additionally, the dendritic eutectic structure and herringbone eutectic structure containing element W occurred, with Fe-enriched matrix phases forming around the eutectic structure (Figure 10d,h).

**Figure 11:**
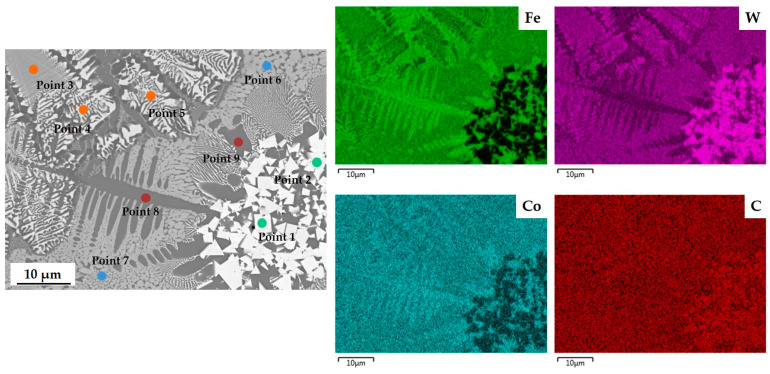
Microstructure and composition results of the cladding layer of specimen 9 (*P* = 5 kW, *S* = 10 mm/s, *R* = 25 g/min).

To identify the element distribution and the phase of the microstructure formed in each laser-cladded specimen, EDS measurements (Figure 11) were performed by enlarging Figure 10h, which shows the most diverse structures and phases, then a semi-quantitative composition was obtained at each point (Table 4). Most of the W element was concentrated in WC particles, and the rest of the W element was evenly distributed except in dendrite regions; this distribution indicates that laser irradiation decomposed some WC particles. Fe and Co elements are present, excluding the areas occupied by WC particles, whereas element C is uniformly distributed throughout the entire region. The compositions of nine points were analyzed (Figure 11 and Table 4). Points 1 and 2 represent decomposed and agglomerated WC particles present in the cladding powder. Points 3, 4, and 5 are carbide phases that contain 50 to 55 at% Fe, 10 to 11 at% W, ~5 at% Co, and ~30 at% C; these correspond to the herring-bone eutectic structure. During the laser cladding process, WC particles decompose into free W and free C atoms as per the following equations [46,47]:

(2) $$2\mathrm{WC}\;\to {\;W}_{2}C+C$$

(3) $$W_{2}C\;\to \;2W+C$$

(4) M+C → MC

(5) $$\mathrm{WC}+W+L\;\to {\;M}_{3}W_{3}C_{2}$$

where M is a metal element such as Fe, Co and Ni, and L is liquid metal. Points 3, 4, and 5, which correspond with the herringbone eutectic structure, are composed of M_3_W_3_C and Fe-Co phases. Points 6 and 7, which are Fe-enriched matrix phases evenly formed in the entire area, are also considered to be M_3_W_3_C phase. This result is consistent with previous studies that explain the formation mechanism of carbide and the growth of eutectic structures containing Fe-Co and carbides [20]. In points 8 and 9, the microstructure that formed in the shape of columnar dendrite is considered to be an Fe-Co alloy phase in which Fe and Co solidified in a liquid state with relatively low W content and high Fe and Co content. Clearly, precipitated phases of the WC-Co composite and its microstructure are associated with decomposition of WC particles and morphology.

### 3.3. Phase Analysis

**Figure 12:**
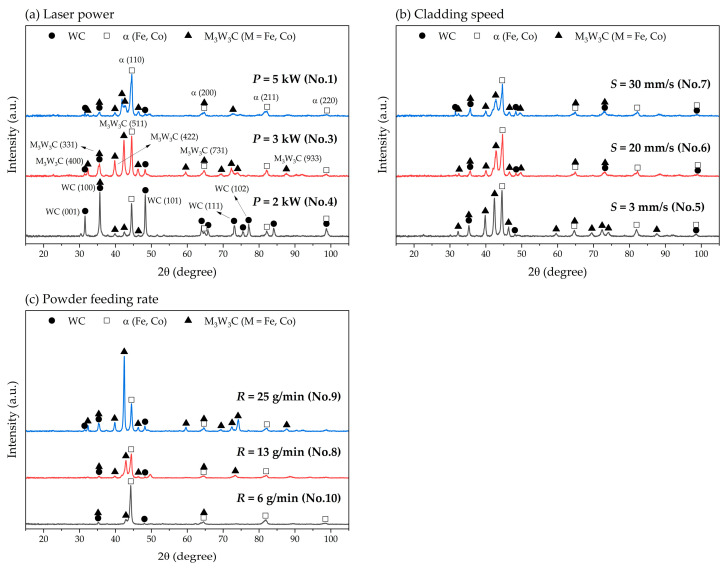
XRD patterns of laser-cladded WC-Co composite coating with various process parameters: (**a**) laser power (*P*); (**b**) cladding speed (S); (**c**) powder feeding rate (*R*).

The phase of the microstructure formed in each laser-cladded specimen was analyzed using XRD measurements of each specimen (Figure 12). Identified phases in each specimen are WC, Fe-Co alloy and M_3_W_3_C (M=Fe, Co) and these results agree with those of previous studies [23,47,48].

As *P* increased, the intensity of WC peaks reduced due to the thermal decomposition of WC particles and the dilution by Fe from the substrate during the laser cladding process. Additionally, the eutectic phase of the M_3_W_3_C and Fe-Co alloy actively occurred and this result shows a similar trend consistent with the changes in microstructure, cladding layer geometry, and dilution rate (Figure 5). As *S* decreased, the proportion of M_3_W_3_C phase increased, with these results being similar to those observed as *P* increased. The XRD peaks of specimens treated at *S* = 20 mm/s and 30 mm/s had similar patterns (Figure 12b), which means that the microstructural evolution did not change significantly (Figure 9c,d). As *R* increased, the M_3_W_3_C phase significantly increased because the number of supplied WC particles increased, promoting the decomposition reaction of WC. The XRD results agree with those of other studies, which indicate that the formation of M_3_W_3_C eutectic carbides improve the bonding strength between WC particle and substrate, and the mechanical property of cladding layer [46].

### 3.4. Mechanical Characteristics

**Figure 13:**
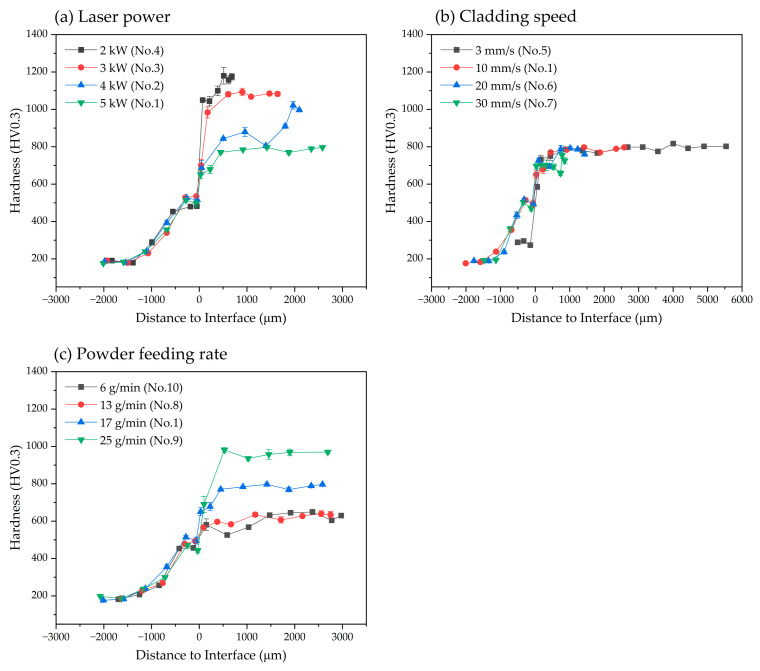
Effect of process parameters on the distribution of microhardness along the cladding-depth direction: (**a**) laser power (*P*); (**b**) cladding speed (*S*); (**c**) powder feeding rate (*R*).

Some process parameters used during cladding affected the measured micro-hardness (Figure 13). The hardness near the substrate was relatively low due to the dilution of the cladding layer by Fe from the substrate.

As *P* increased, the hardness of the cladding layer decreased (Figure 13a). The amount of Fe supplied by melting of the substrate increased and the Fe dissolves in the cladding layer. This result leads to an increase in the formation of Fe-Co alloy phases, which have lower mechanical properties than carbide phases in the cladding layer (Figure 5 and Figure 8). Change in *S* did not affect the hardness of the cladding layer (Figure 13b). As *S* increased, *D* remained constant (Figure 5b). The constant *D* occurs because an increase in *S* does not significantly change the mass fraction of the Fe-Co alloy phase, even though the herringbone structure (i.e., the eutectic phase formed by M_3_W_3_C and the Fe-Co alloy phase) is suppressed. Increase in *R* resulted in decrease in formation of the W-depleted and Fe-enriched columnar dendrite, and a decrease in the mass fraction of dendrites. Concurrently, the content of dendritic eutectic structure and herringbone eutectic structure increased, and the micro-hardness of the cladding layer increased.

Among the 10 process conditions (Table 2), specimens 3 and 9, with low *D*, showed excellent hardness, with an average HV_0.3_ of 1013 for specimen 3, and 917 for specimen 9. In addition, these specimens did not have the Fe-Co alloy phase and its dendrites. However, pores and holes were formed in these specimens (Figure 4), which were attributed to the insufficient melting of cladding powders, which was a consequence of low *P* or high *R* [49,50]. Due to the poor fluidity of the molten pool, insufficient melting results in trapped gas and the generation of pores in the molten cladding layer.

To increase the hardness of the cladding layer, the formation of phases and microstructure that have lower mechanical properties than the supplied cladding material should be prevented, and *D* should be kept low. In contrast, irregular and porous layers, such as those observed in specimen 3, with relatively low laser power, occasionally exhibit excellent hardness. Thus, in order to achieve a dense and uniform cladding layer with excellent mechanical properties, the process parameters and conditions should ensure sufficient melting of the cladding material. Additionally, the formation of the metal-alloy phase diluted by the substrate should be suppressed, while the formation of the carbide phase and microstructure should be encouraged.

### 3.5. Effect of Powder Type on the Microstructure and Wear Resistance

**Figure 14:**
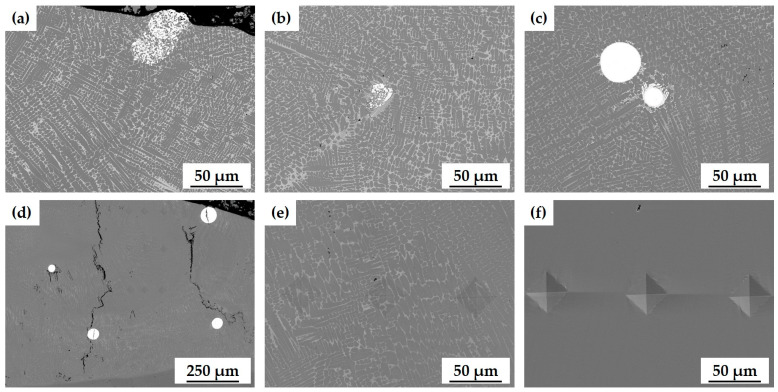
Microstructure of cladding layer with various powder types: (**a**) powder A (WC-12Co, Metco); (**b**) powder B (WC-12Co, HiMC); (**c**–**e**) powder C (WC-30Ni); (**f**) powder D (Ni-Cr alloy).

The various powder types caused different microstructures of the cladding layer even when treated with the same laser cladding parameters (Figure 14). When powders A and B (primarily WC-Co) were used, WC particles were partially or sufficiently decomposed due to thermal damage by the laser irradiation (Figure 14a,b). When powder C (primarily WC-Ni) was used, WC particles were not decomposed and retained their spherical shape (Figure 14c), and cracks formed vertically from the cladding layer throughout the entire region, including around WC particles (Figure 14d). The Ni-17Cr alloy powder formed the densest layer, with no variation in the microstructure over the entire area (Figure 14f).

During solidification of cladding materials, tensile stress is applied to WC particles due to the contraction of liquid metal. Consequently, cracks are prone to initiate at stress-concentrations, which are points associated with these WC particles, and then propagate along the maximum stress direction within the particles [51,52]. In powder C, WC particles were larger than in powders A and B. The cracks tended to traverse these large WC particles because they are more predisposed than small particles to contain defects, and material defects serve as stress concentration points which initiate the formation of cracks [52,53].

**Figure 15:**
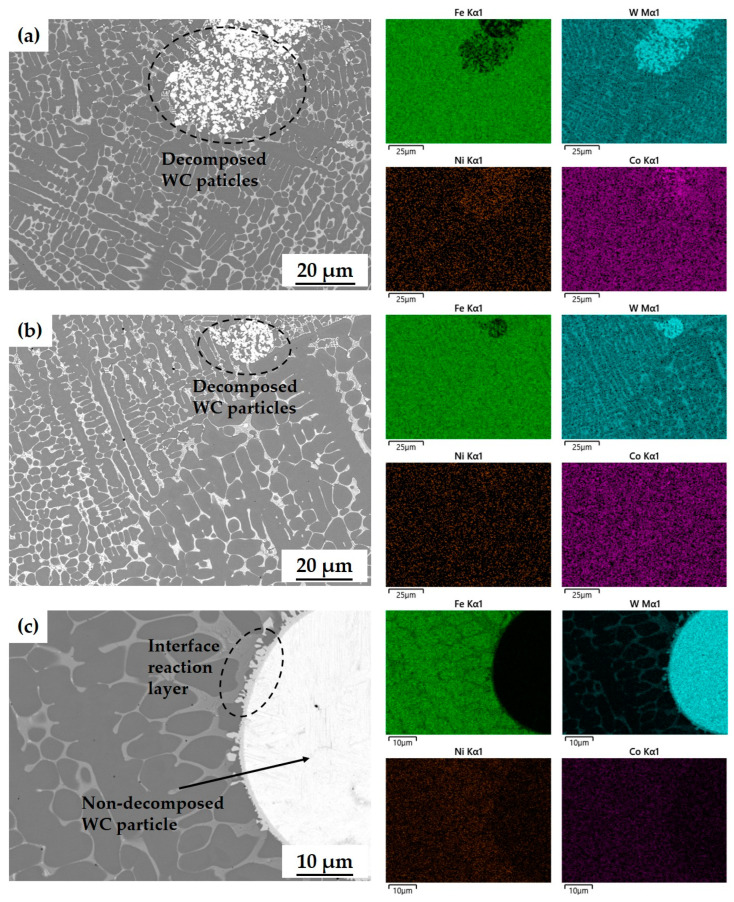
EDS results of cladding layer with various powder types: (**a**) powder A (WC-12Co, Metco); (**b**) powder B (WC-12Co, HiMC); (**c**) powder C (WC-30Ni).

The distributions of elements around WC particles differed among powder types (Figure 15). Dissolution and thermal decomposition of WC particles results in precipitation of different carbides, depending on the size of the WC particles and the processing parameters [54]. When WC-Co powder was used, WC particles were initially relatively small, and broke into small pieces and dissolved sufficiently when heated. In contrast, WC-Ni powders have relatively large WC particles that did not decompose, resulting in the formation of an interface reaction layer around them (Figure 15c). Compared with the WC-Co system, the WC-Ni system has a narrower range in which liquid and WC coexist, so use of WC-Ni powder suppresses the diffusion of W and the decomposition of WC particles [55].

In WC-Co powders A and B, the W element that was released by the decomposition of WC was distributed throughout the matrix that was composed of Fe and Co, and Fe also penetrated the WC particles (Figure 15a,b). In contrast, in WC-Ni powder C, W was not distributed in the Fe-Ni alloy phase, and Fe did not penetrate the WC particles (Figure 15c). Despite the four powders having different powder particle sizes, it is not the size of the powder particles that influences the microstructure of the laser cladding layer and the decomposition of WC, but rather the size of the initial WC particles contained within the powder particles and the alloying elements such as Co and Ni that constitute the matrix phase.

**Figure 16:**
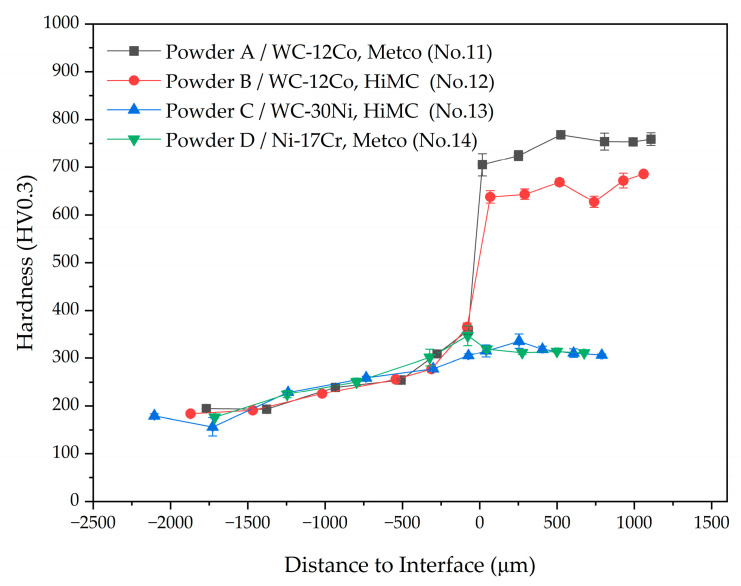
Distribution of microhardness along the cladding depth direction with various powder types.

The microhardness of the cladding layer varied depending on the powder types (Figure 16). The cladding layer composed of WC-Co powders had the highest microhardness under the same laser cladding process parameters, whereas layers composed of WC-Ni powders had low microhardness, similar to those of layers composed of Ni-Cr alloy powder, despite containing WC particles.

The difference in the hardness of WC-Co and WC-Ni powders is that W elements within WC-Co powders are widely distributed, both in the hard phase, such as M_3_W_3_C carbide (Figure 15a, light gray), and in Fe-alloy phase (Figure 15a, dark gray). However, the W element did not appear in the Fe-alloy phase within layers composed of WC-Ni powder (Figure 15c, dark gray). Additionally, formation of a W-depleted Fe alloy phase was promoted more in the WC-Ni powder than in the WC-Co powder (Figure 15c).

These results indicate that the formation of carbides such as M_3_W_3_C, and the distribution of W into an Fe-alloy phase have significant effects with which to increase the microhardness of the WC-Fe-Ni composite coating when the WC particles are completely decomposed in the cladding layer.

**Figure 17:**
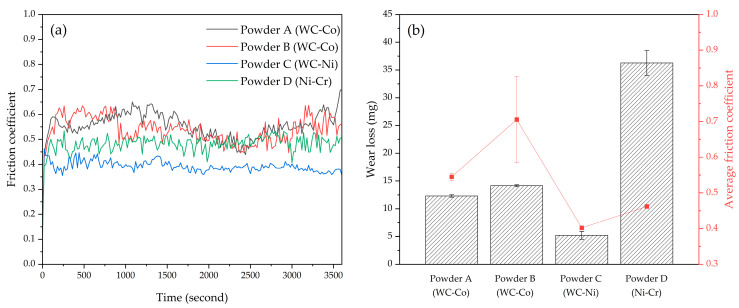
Wear-resistance test results with various powder types: (**a**) plot of friction coefficient; (**b**) wear loss and average friction coefficient (conditions: rotation speed, 1000 rpm; load, 300 N; duration, 3600 s; temperature, 298 K).

The wear resistance results of the specimens fabricated by laser cladding were compared for powders A, B, C, and D using the same conditions (rotation speed: 1000 rpm, load: 300 N, duration: 3600 s, temperature: 298 K) (Figure 17). The WC-Co coatings (powder A and powder B), which formed the cladding layer with high hardness, exhibit a relatively high friction coefficient and wear loss, whereas WC-Ni coatings (Powder B) have the lowest friction coefficient and lowest wear loss. Wear resistance of the composite coatings is largely dependent on the microstructure, phase distribution, and mechanical properties of cladding layer [56], and the increased wear resistance of the cladding layer that contained WC particles is caused by their improved microhardness [57].

**Figure 18:**
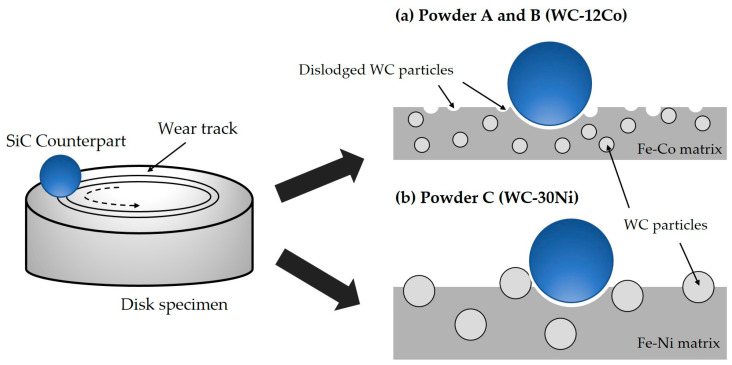
Schematic diagram of the wear process: (**a**) powders A and B (WC-12Co) and (**b**) powder C (WC-30Ni).

However, cladding layers of powder C had superior (i.e., lower) wear loss and higher average friction coefficient compared with those of powder A and powder B, despite the microhardness of the cladding layer for powder A and powder B being much higher than that of powder C. The hard WC-Co composites typically exhibit lower toughness compared with WC-Ni composites [58,59]. Furthermore, the enhancement in hardness with the decrease of WC grain size can lead to a sharp drop in the toughness of the composites [60]. Consequently, fine WC particles embedded in a relatively hard Fe-Co matrix phase, such as M_3_W_3_C phase, as observed in the cladding layers of powder A and B, are susceptible to detachment from the matrix during wear processes (Figure 18a). In contrast, coarse WC grains within an Fe-Ni matrix phase are capable of withstanding repeated friction loads over extended periods (Figure 18b). When the cladding layer comprises a hard Fe-Co matrix and WC particles, as in powders A and B, the wear properties could deteriorate due to the propensity of fine WC particles within a hard matrix phase to dislodge from the matrix. During the wear process, coarse WC particles within powder C have a strong effect on the suppression of sliding friction, so the wear resistance increases [18]. On the contrary, cladding layers of Ni-Cr powder (powder D) exhibited a low friction coefficient and high wear loss.

**Figure 19:**
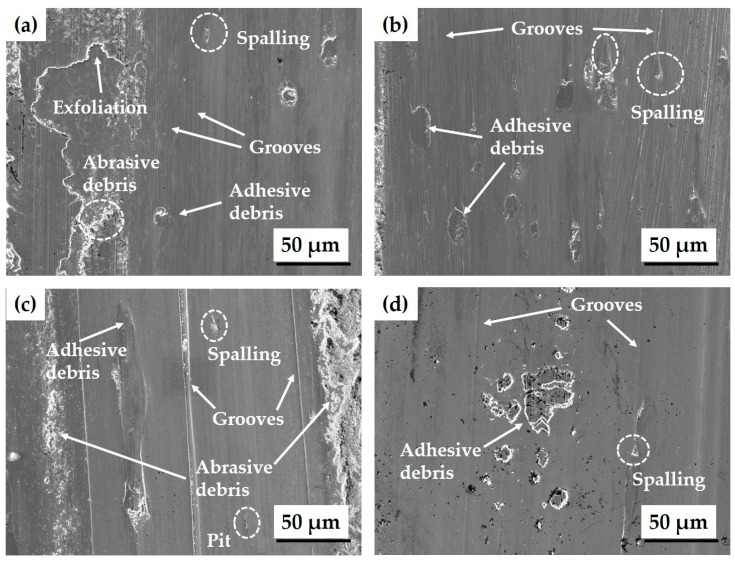
The wear surface morphology of the composite coatings: (**a**) powder A (WC-12Co, Metco); (**b**) powder B (WC-12Co, HiMC); (**c**) powder C (WC-30Ni); (**d**) powder D (Ni-Cr alloy).

To identify the wear mechanism of composite coatings, the worn morphology of each coating was analyzed (Figure 19). The worn surfaces of cladding layers produced using powders A (WC-12Co, Metco) and B (WC-12Co, HiMC) had similar features, such as parallel grooves, exfoliation, and debris (Figure 19a,b). These observations suggest a simultaneous occurrence of abrasive and adhesive wear. The protruding coating layer comes into contact with the counter-grinding ball, and excessive local stress leads to points of contact adhering or welding to each other. These points move tangentially with each other and so shear off in a process that results in material peeling and adhesive wear [40]. Additionally, the presence of the hard WC phase contributes to the formation of shallow and narrow wear grooves on the surface, traits which indicate abrasive wear. In the case of powders A and B, the wear loss was relatively large due to the predominance of adhesive wear, which was in turn caused by the loss of fine WC particles and the shearing of the matrix phase with a rough surface, rather than the grinding action of hard WC particles and the M_3_W_3_C phase.

For powder D (Ni-Cr alloy), the worn surface morphology shows a combination of adhesive and abrasive wear, similar to the worn surfaces of layers produced using powder A (WC-12Co, Metco) or B (WC-12Co, HiMC) (Figure 19d). However, the wear resistance results for Ni-Cr alloy coatings differ from those of WC-Co coatings (Figure 17). Powder D (Ni-Cr alloy) shows significant wear mass loss, primarily attributed to the absence of hard materials such as WC particles. In contrast, the friction coefficient is comparatively low due to the good wear resistance of Ni-Cr alloy coatings when wear occurs due to thermal stress [61,62].

The worn surface of powder C (WC-30Ni) coating exhibits grooves, spalling, and debris (Figure 19c). These traits indicate that adhesive wear and abrasive wear occur alternately. However, the presence of relatively deep grooves and a substantial amount of debris suggests that abrasive wear predominates. As the counter-grinding ball slides, the disparity in thermal expansion coefficients between the WC particles and the Ni binder metal results in the bonding force at the interface being weaker than the residual stress [63]. When coarsened WC particles detach from the matrix, this detachment induces micro cutting, the formation of pits, and the growth of deep grooves, thereby intensifying abrasive wear. As a result, the predominance of abrasive wear leads to a reduction in wear loss. 

Hard materials, including WC particles, have excellent wear resistance. The effectiveness of WC particles in the cladding layer depends on the relationship between the wear mechanisms and the mechanical properties, which are related to the microstructure of the cladding layer [64]. The increased wear resistance of powder C (WC-30Ni) is ascribed to the existence of non-decomposed coarse WC particles and an abrasive wear mechanism [57,65]. When the cladding layer contains thermally decomposed WC particles with a fine particle size (e.g., powders A and B), the increase in hardness is primarily attributed to variations in the distribution of W and an increase in carbide phases (e.g., WC, M_3_W_3_C) within the coating layer. However, wear resistance tends to deteriorate due to the predominance of the adhesive wear mechanism, which results from the separation of fine WC particles from the matrix rather than from the effects of the carbide phase formed by the decomposition of WC particles. Therefore, it is important to select the appropriate powder system and particle size to reflect the purpose of the coating. Additionally, optimizing cladding parameters and controlling the microstructure of the cladding layer could enhance the mechanical properties and wear resistance, thereby solving the problems related to phase distribution, deterioration of cladding layer properties, and product quality in laser cladding.

## 4. Conclusions

This paper reports an investigation into the way in which laser cladding process parameters and powder types affect the microstructure and mechanical properties of WC-Co-Ni composite coating for continuous caster rolls. Our analysis considered the effects of laser power *P*, cladding speed *S*, and powder feeding rate *R* on the geometry, microstructure, and hardness of the cladding layer. Then the mechanical properties and wear resistance of specimens that had been produced with each WC-Co composite, WC-Ni composite, and Ni-Cr alloy powders with the same process parameter were evaluated. The conclusions are as follows.

(1) As *P* increased, *D* of the cladding layer increased. Fe from the substrate diluted the cladding layer by dissolving into it, and the proportion of Fe-Co alloy phases increased in the cladding layer.
(2) As *S* increased, the hardness and dilution rate of the cladding layer did not change. The mass fraction of the Fe-Co alloy phase remained constant, despite the suppression of the herringbone structure, which is the eutectic phase formed by the M_3_W_3_C and Fe-Co alloy phases.
(3) As *R* increased, the formation of W-depleted and Fe-enriched columnar dendrite decreased, and the mass fraction of dendrite decreased. Concurrently, dendritic eutectic structure and herringbone eutectic structures grew, and the micro-hardness of the cladding layer increased.
(4) To improve the hardness of the cladding layer in the WC-Co-Ni composite coating, the formation of Fe-Co alloy phases, which have lower mechanical properties than the carbide phases (e.g., WC, M_3_W_3_C), should be prevented, and the dilution rate of the cladding layer should be minimized.
(5) The microstructure and hardness of the laser cladding layer depend not only on the process parameters, but also on powder characteristics, such as WC particle size and the type of binder material.
(6) Depending on the WC particle size and the characteristics of the binder metal within the cladding layer, wear behavior can differ significantly in cladding layers of each powder. To enhance the wear resistance of the laser cladding coating layer, it is essential to consider the wear mechanism, which encompasses both the hardness and the characteristics of the powder used.

## Figures and Tables

**Table 1:** Chemical compositions and characteristics of laser cladding powders.

Powder	Composition (wt%)	Supplier	Characteristic
A	W(bal.), Co(10.5~13.5), C(5.0~5.8), Fe(0.2)	Metco	Co-added
B	WC(88), Co(12)	HiMC	Co-added
C	WC(69), Ni(28), (B-Cr-Si-Fe)(3)	HiMC	Ni-added
D	Cr(17), Fe(4), Si(4), B(3.5), C(1), Ni(bal.)	Metco	Ni-based

**Table 2:** Laser cladding parameters for individual specimen using powder A.

Powder	Specimen No.	Laser Power (kW)	Cladding Speed (mm/s)	Powder Feed Rate (RPM (g/min))	Off-Set (mm)	Optic (Φ)	No. of Track
A	1	5	10	1.3 (17)	-	5.5	1
	2	4	10	1.3 (17)	-	5.5	1
	3	3	10	1.3 (17)	-	5.5	1
	4	2	10	1.3 (17)	-	5.5	1
	5	5	3	1.3 (17)	-	5.5	1
	6	5	20	1.3 (17)	-	5.5	1
	7	5	30	1.3 (17)	-	5.5	1
	8	5	10	1.0 (13)	-	5.5	1
	9	5	10	2.0 (25)	-	5.5	1
	10	5	10	0.5 (6)	-	5.5	1

**Table 3:** Laser cladding parameters for wear resistance test using various powders.

Powder	Specimen No.	Laser Power (kW)	Cladding Speed (mm/s)	Powder Feed Rate (RPM (g/min))	Off-Set (mm)	Optic (Φ)	No. of Track
A (WC-12Co, Metco)	11	3	20	0.5 (6)	2.5	5.5	5
B (WC-12Co, HiMC)	12						
C (WC-30Ni)	13						
D (Ni-Cr alloy)	14						

**Table 4:** Element distribution at each marked point of specimen 9 in Figure 11.

Point	Morphology	Chemical Composition (Atomic %)					
		C	Cr	Fe	Co	Ni	W
1	Faceted	64.40	0.02	2.16	0.32	0.08	33.02
2	Faceted	64.54	0.00	1.69	0.22	0.04	33.51
3	Herringbone eutectic	32.50	0.06	52.23	4.84	0.04	10.33
4	Herringbone eutectic	32.52	0.06	50.55	5.01	0.14	11.71
5	Herringbone eutectic	29.03	0.09	55.01	5.48	0.08	10.30
6	Refined grain	37.84	0.10	46.13	4.15	0.09	11.69
7	Refined grain	34.04	0.14	50.57	4.72	0.15	10.38
8	Columnar dendrite	26.70	0.07	62.61	6.40	0.13	4.10
9	Dendrite	27.43	0.06	61.71	6.69	0.15	3.95

## Data Availability

The data presented in this study are available on request from the corresponding author. The data are not publicly available due to the need for consultation between POSTECH and SungWook Co., Ltd. regarding data availability.

## Nomenclature

**Variable**	**Definition**	**Unit**
*D*	Dilution of cladding layer	proportion
*f*	Friction coefficient	Proportion
*h*	Height of cladding layer	mm
*P*	Laser power	kW
*R*	Powder feeding rate	g/min
*S*	Speed of laser scan (cladding speed)	mm/s
*w*	Width of cladding layer	mm
